# Bacterial and Fungal Dynamics During the Fermentation Process of *Sesotho*, a Traditional Beer of Southern Africa

**DOI:** 10.3389/fmicb.2020.01451

**Published:** 2020-06-30

**Authors:** Errol D. Cason, Bokang J. Mahlomaholo, Matšepo M. Taole, George Ooko Abong, Jan-G Vermeulen, Olga de Smidt, Marcele Vermeulen, Laurinda Steyn, Angel Valverde, Bennie Viljoen

**Affiliations:** ^1^Department of Microbial, Biochemical and Food Biotechnology, University of the Free State, Bloemfontein, South Africa; ^2^Department of Animal, Wildlife, and Grassland Sciences, University of the Free State, Bloemfontein, South Africa; ^3^Department of Biology, National University of Lesotho, Maseru, Lesotho; ^4^Department of Food Science, Nutrition and Technology, University of Nairobi, Nairobi, Kenya; ^5^Centre for Applied Food Sustainability and Biotechnology (CAFSaB), Central University of Technology, Bloemfontein, South Africa

**Keywords:** *Sesotho*, Traditional Fermented Beer, fermentation, Microbial patterns, next generation sequencing, Bacterial diversity, fungal diversity

## Abstract

*Sesotho* is an indigenous cereal-based fermented drink traditionally produced in the mountain kingdom of Lesotho, Southern Africa. The present study sought to examine the microbial (bacterial and fungal) community composition of *Sesotho* at five fermentation stages in five different locations. Using culture-independent (Illumina sequencing) techniques it was found that the bacterial communities followed similar successional patterns during the fermentation processes, regardless of geographical location and recipe variation between breweries. The most abundant bacterial taxa belonged to the phyla Firmicutes (66.2% of the reads on average) and Proteobacteria (22.1%); the families Lactobacillaceae (54.9%), Enterobacteriaceae (14.4%) and Leoconostrocaceae (8.1%); and the genera *Lactobacillus* (54%), *Leuconostoc* (10.7%), *Leptotrichia* (8.5%), and *Weissella* (5.5%). Most fungal taxa were from the phyla Ascomycota (60.7%) and Mucoromycota (25.3%); the families Rhizopodaceae (25.3%), Nectriaceae (24.2%), Saccharomycetaceae (16%) and Aspergillaceae (6.7%); and the genera *Rhizopus* (25.3%), *Saccharomyces* (9.6%), and *Aspergillus* (2.5%). Lactic acid bacteria (LAB) such as *Enterococcus*, *Pediococcus*, *Lactobacillus*, *Leuconostoc*, and *Wiesella*; as well as yeasts belonging to the genus *Saccharomyces*, were dominant in all breweries during the production of *Sesotho*. Several pathogenic and food spoilage microorganisms (e.g., *Escherichia*, *Shigella*, *Klebsiella*, etc.) were also present, but the study demonstrated the safety potential of the *Sesotho* fermentation process, as these microbial groups decline throughout *Sesotho* production. The functional profiles of the different brewing steps showed that the process is dominated by chemoheterotrophic and fermentative metabolisms. This study reveals, for the first time, the complex microbial dynamics that occur during *Sesotho* production.

## Introduction

Fermented foods and beverages are worldwide consumed on a daily basis, representing a large portion of human diets (Achi, 2005; Marshall and Mejia-Lorio, 2012). In general, these spontaneous fermentations start-off with highly diverse microbial communities consisting of bacteria, yeasts and molds that are introduced directly from the raw materials, utensils, or the producers themselves (Minervini et al., 2014; Achi and Ukwuru, 2015). For instance, the initial stages of the fermentation of cereal-based fermented foods and beverages predominantly consist of lactic acid bacteria (LAB). These bacteria produce lactic acid, acetic acid, carbon dioxide, and ethanol, which influence the biochemical dynamics within the substrate (Steinkraus, 2002). This in turn influences the physio-chemical parameters (such as pH) as well as sugar and salt concentrations of the final products (Bigot et al., 2015; Tamang et al., 2016, 2020). The continuous fluctuation in these physio-chemical properties creates stress conditions that reduce microbial diversity over time, leading to the emersion of the dominant microbiota (Muyanja et al., 2003; Assohoun-Djeni et al., 2016; Escobar-Zepeda et al., 2016; Ruiz Rodríguez et al., 2016). In addition, LAB produce antibacterial agents such as bacteriocins that directly inhibit pathogenic- and food spoilage-bacteria (Ahmad et al., 2016; Vera-Pingitore et al., 2016). Therefore, these microbial communities strongly influence the sensory quality, nutrient availability as well as safety and longevity of these products (Hwanhlem et al., 2014; Martinez et al., 2015; Chuah et al., 2016). This, coupled to their potential biotechnological applications (Park et al., 2012), has led to increased interest in the microbial consortia associated with traditional fermented food and beverages. Furthermore, many of these products are only produced locally using undocumented traditional techniques. A such, it is important to document these traditional processes in order to preserve this indigenous knowledge for future generations.

The complex microbial consortia associated with spontaneous fermented foods often contain uncultivable microorganisms, which share similar physiological properties and contribute to similar food characteristics, but are phylogenetically distant species (Bigot et al., 2015). As a result, culture-dependent methods do not give a comprehensive picture of the microbial diversity of traditionally fermented food and beverages. In contrast, culture-independent methods, such as Next Generation Sequencing (NGS) and Metagenomics, allow study of the microbial communities without the need for isolation and additional laboratory cultivation of individual species (Morozova and Marra, 2008; Kergourlay et al., 2015; Escobar-Zepeda et al., 2016). More specifically, targeted NGS sequencing of the 16S rRNA genes and ITS rDNA regions has become the new standard to profile bacterial and fungal communities, respectively. This methodology has recently been used for describing the microbial profile of a few traditional fermented foods (Kergourlay et al., 2015; Escobar-Zepeda et al., 2016; Tamang et al., 2016, 2020).

*Sesotho* is a popular spontaneously fermented beer produced in Lesotho (Southern Africa), prepared from milled maize, sorghum or wheat flour (sometimes a mixture of these flours). *Sesotho* beer is turbid, has a thin consistency and a distinctive sour taste. It is mostly prepared at village-level for small scale commercial purposes, but it is also prepared as an inebriating drink at funerals, marriages, and other cultural ceremonies. However, there is currently no information available on the microbial diversity of this traditional fermented beverage.

The aim of this study is to characterize the microbial diversity (bacterial and fungal) present during the five stages of *Sesotho* fermentation, at five distinct locations in Lesotho using NGS techniques.

## Materials and Methods

### Sample Collection

**FIGURE 1:**
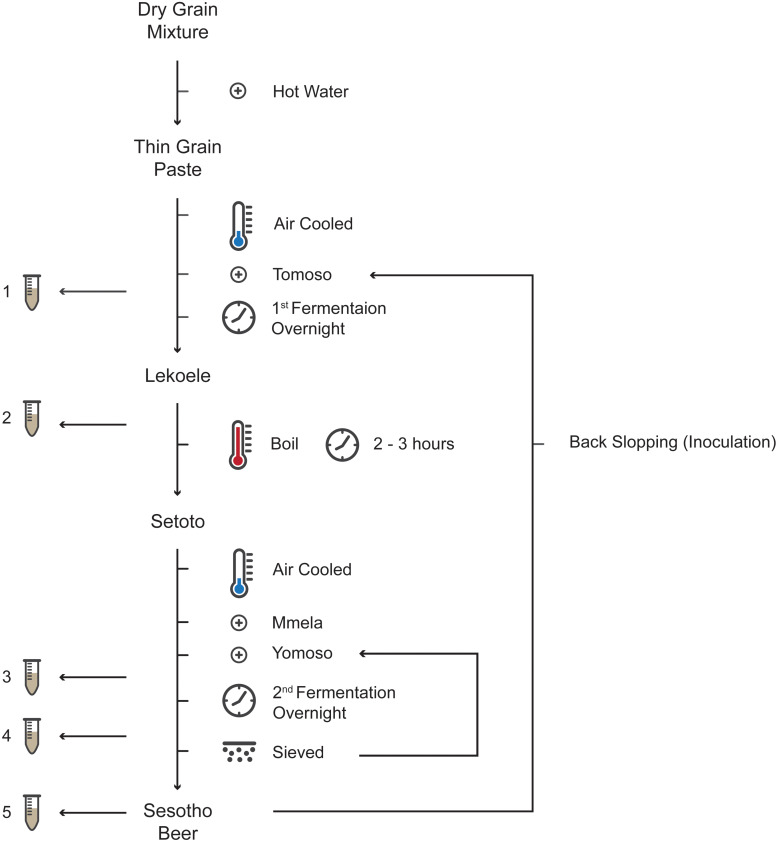
A flow diagram of *Sesotho* preparation and sampling points (1–5).

The microbiology and physicochemistry of *Sesotho* was investigated at five districts (breweries) in Lesotho, namely, Maseru (West), Mafeteng (South), Thaba-Tseka (Central), Butha-Buthe (North), and Mokhotlong (East; Supplementary Figure 1 and Supplementary Table 1), at five stages during the fermentation process (Figure 1). The first sample (1) was taken an hour after the addition of the first starter culture to initiate the first fermentation phase. The second sample (2) was taken after the first fermentation phase (at approximately 8 h of fermentation). The third sample (3) was taken an hour following the addition of the second starter to initiate the second and final fermentation phase. The fourth sample (4) was taken prior to sieving the beer (separating the sorghum malt from the beer), approximately 8 h after the second fermentation. The fifth sample (5) was taken from the final product, approximately 8 h into maturation. Samples were aseptically collected in 1 L sterile plastic bottles and immediately stored at 4°C. Samples were transported, within 48 h, to the Department of Microbial, Biochemical and Food Biotechnology at the University of the Free State (UFS) where they were stored at −20°C until genomic DNA extraction and physicochemical analysis were performed. A total of 25 samples were collected, one at each of the identified brewing steps from five different breweries. Due to the nature of the traditional brewing industry (traditional brewers do not have the facilities to brew more than one batch at a time), biological replicates within breweries could not be obtained.

### Physio-Chemical Analysis

The pH of each sample was measured on-site using a HANNA^®^ pocket pH meter (HANNA, United States). Acetate, lactate, glucose and glycerol were analyzed on a Finnigan Surveyor Plus HPLC fitted with a Biorad Aminex HPX 87H ion exchange column (300 mm × 10 mm), using 5 mM sulphuric acid at a flow rate of 0.6 ml min^–1^ as eluent. Acids were detected with a PDA detector at 202 nm and glycerol and glucose with a refractive index detector connected in series. Analytes were quantified by external standards. Prior to HPLC analysis, 5 ml of each of the samples were centrifuged (10,000 × *g* for 5 min) and the supernatant collected for analyses. Ethanol concentrations were determined by gas chromatography (Shimadzu GC-2010 Pro), carrier gas: hydrogen 60 cm.s^–1^,CPWax 52CB column [30 m (L) × 0.32 mm (ID) × 0.25 μm (Film thickness)]. The flame ionization detector temperature was 260°C and the injection port was 150°C. Injection volume was 1 μL with a split of 1:10. The initial column temperature was 40°C held for 3 min and then ramped to 256°C at 25°C min^–1^ and held for 1°min. Shimadzu GC Solution software was used for instrument control, data collection and analysis.

### 16S rRNA Amplicon Sequencing

Following thawing, a 5 ml fraction of each sample was centrifuged using a Beckman centrifuge J2–21 (Beckman, United States; 8,000 *× g* for 5 min) and the supernatant was discarded. The remaining pellet was washed twice with TE buffer (100 mM Tris–EDTA, pH 8) following centrifugation (8,000 *× g* for 5 min). The washed pellets were re-suspended in 5 ml of TE buffer as above. The pellets were frozen at −80°C and transported on dry ice to the Centre of Proteomics and Genomic Research (CPGR) in Cape Town, South Africa, for genomic DNA extraction, library preparation and sequencing. Briefly, the sequencing library was obtained by amplifying a ∼550 bp region located in the hypervariable V3/4 region of the 16S rRNA gene using region of interest-specific primers with overhang Illumina adapter overhang nucleotide sequences (16S BacF 5′ – TCGTCGGCAGCGTCAGATGTGTATA-AGAGACAG CCTAC GGGNGGCWGCAG – 3′ and 16S BacR 5′ – GTCTCGTGG GCT-CGGAGATGTGTATAAGAGACAGGACTACHVGGGTA TCTAATCC – 3′; Klindworth et al., 2013). The 16S V3/4 amplicons were purified using the Agencourt AMPure XP bead clean up kit (Beckman Coulter Genomics, Danvers, MA, United States), followed by a second amplification to attach dual indices and Illumina sequencing adapters using the Nextera XT Index kit (Illumina, San Diego, CA, United States). Final purification using the AMPure XP bead clean up kit (Beckman Coulter Genomics) was done, followed by library quantification, normalization, pooling and denaturing before being subjected to 2 × 300 cycle sequencing on the Illumina MiSeq using the MiSeq v3 reagent kit (Illumina). The raw data from each of the sequenced samples were submitted to the Sequence Read Archive (SRA) of the NCBI, under Project ID PRJNA605088.

### Fungal ITS Amplicon Sequencing

Samples were thawed and a 50 ml fraction of each sample was centrifuged (Beckman centrifuge, Algera^TM^ 25R; Beckman, United States; 5,000 × *g* for 5 min) and the supernatant was discarded. This was repeated three times. The obtained pellets (500 μl) were transferred to a 2 ml microcentrifuge tube and DNA was extracted according to Moller et al. (1992). DNA concentrations were determined on a NanoDrop 2000 (Thermo Scientific, NanoDrop products, Wilmington, DE, United States) and standardize to 100 ng.μl^–1^ in nuclease free water (WhiteSci, Whitehead Scientific, Cape Town, South Africa) prior to shipment to MR DNA (www.mrdnalab.com; Shallowater, TX, United States). Fungal communities sequencing libraries were obtained by amplification of the internal transcribed spacer 1 (ITS1) region using ITS1 and ITS2 primers with Illumina adapter overhang nucleotide (ITS1 5′– TCGTCGGCAG-CGTCAGATGTGTATAAGAGACAGTCCGTAGGTGAACCTG CGG – 3′ and ITS2 5′– GTCTCGTGGGCTCGGAGATGT GTATAAGAGACAGGCTGCGTTCTTCATCGATGC – 3′ (White et al., 1990). Resulting ITS1 amplicons were purified using calibrated AMPure XP beads (Beckman Coulter, Inc., Pasadena, United States) and was then used to prepare DNA library following Illumina Truseq DNA library preparation protocol. Paired-end sequencing (2 × 300) was performed on the Illumina MiSeq sequencing platform (Illumina, San Diego, United States) at MR DNA (www.mrdnalab.com; Shallowater, TX, United States). The raw data from each of the sequenced samples were submitted to the SRA of the NCBI, under Project ID PRJNA605302.

### Bioinformatic Processing

Sequence analysis and bioinformatic processing was performed according to Cason et al. (2017). Briefly. quality control of the obtained 16S rRNA and ITS sequence data was performed using PrinSeq-lite v0.20.4 (Schmieder and Edwards, 2011). All data sets were pre-processed and trimmed to obtain an average quality score ≥ 20 using a 5 nt window with a 3 nt step. All sequences shorter than 200 bp were filtered out. Paired end reads were merged using PEAR 0.9.6 (Zhang et al., 2014). Quality reads were analyzed using QIIME v1.9.1 as described by Caporaso et al. (2011). Briefly, the demultiplex and quality filtering script in QIIME was run with default parameters to obtain a FASTA output file. Chimeric sequences were identified with the identify_chimeric_seqs.py command using Usearch v6.1.544 (Edgar, 2010) against the RDP “Gold” database (Edgar, 2010) for bacteria/archaea and the UNITE database (Abarenkov et al., 2010) for fungi. Chimeric sequences were filtered out using the filter_fasta.py command. Operational Taxonomic Unit (OTU) picking and taxonomic affiliations of the representative OTUs were carried out using the pick_open_reference_otus.py script, at 97% sequence identity against the SILVA 132 database (Quast et al., 2013) for the bacterial/archaeal 16S rRNA and the UNITE (Abarenkov et al., 2010) for the fungal ITS data, respectively.

### Data Analysis

The core microbiome (present in 100% of the samples) were identified using the compute_core_microbiome.py command in QIIME. Potential functional capacity of the bacterial OTUs were predicted using FAPROTAX v1.2.2 (Louca et al., 2016). FAPROTAX is a database that maps prokaryotic taxa to functional characteristics available using current information on cultured strains. The annotated OTU table was matched with the species information in the database using the “collapse_table.py” command. The relative abundances of the functional groups in each sample was calculated as the cumulative abundance of OTUs assigned to each functional group. Analysis of the abundance tables were carried out using R v3.6.1^1^ (R Core Team, 2013) and the phyloseq package (McMurdie and Holmes, 2013). OTU tables were rarefied to 77 370 (bacteria) and 43 (fungi) reads per sample. Richness and phylogenetic diversity (PD) were calculated using the package picante (Kembel et al., 2010) in R. To test for differences in chemistry, richness and PD between different breweries and brewing steps, a Kruskal–Wallis test followed by a Bonferroni-adjusted *post hoc* Wilcoxon signed-rank test was used. Both tests and Bonferroni corrections were conducted with functions “kruskal_test,” “wilcox_test,” and “adjust_*p* value” in the rstatix package (Kassambara, 2020) in R. Plots were visualized using the ggplot2 package (Wickham, 2016). For beta diversity analysis the taxonomic structures of the microbial communities were visualized using principal coordinate analysis (PCoA) with normalized unweighted UniFrac distances for bacteria and the Bray–Curtis distance metric for fungi. To asses beta diversity differences between different breweries and brewing steps a permutational analysis of variance (PERMANOVA) was used. These analyses were performed with the “adonis” functions in vegan (Oksanen et al., 2019) for R.

## Results and Discussion

### Brewing Process of Sesotho (Traditional Methods and Beliefs)

In general, *Sesotho* is produced from either milled-maize, -sorghum or -wheat. However, a mixture of these grains are often used depending on the availability as well as brewer’s preference. The preparation of *Sesotho* (Figure 1) involves the mixing of maize or sorghum flour with wheat flour by hand. Warm water is then mixed with the flour to form a thick paste, followed by addition of boiling water to form a thinner paste. The resulting thin paste is then cooled before Tomoso (liquid starter obtained from the previous successful batch of the initial fermentation) is added. The amount of starter added varies greatly from household to household as it depends on its perceived strength (based on its sourness of taste) and the amount of beer that is being produced. The vessel is covered and left to ferment overnight to form Lekoele. Fermentation length is highly dependent on starter strength (quantity and viability) and temperature (fermentation takes longer during the winter months). The Lekoele is then cooked and the liquid upper phase transferred to a pot and boiled. The remaining liquid and solid phases are then mixed by slowly stirring and poured into the boiling upper phase liquid. Following boiling (2–3 h), the mixture attains a thick consistency and is called Setoto. The Setoto is left to cool at which point Mmela (sorghum malt) and Yomoso (also known as Kokola or Moroko, the spent solid starter obtained from the previous successful batch) are added. The vessel is then covered and left fermenting overnight. Following fermentation, the mixture is sieved thoroughly prior to consumption (Supplementary Figure 2). Brewers usually add sugar to the finished product with the belief that it will produce a more potent beer. Rural communities often also burn a piece of paper over the brew. While burning it is moved over the brew in a circular motion until it dies out and the ashes are dropped into the brew. This ritual smoking process is called “*Ho cheseletsa*” and is meant to encourage a smooth and successful fermentation process.

### Bacterial Diversity and Abundance

A total of 9 885 bacterial OTUs (97% similarity cut-off), ranging from 600 to 2 543 OTUs per sample, were found in the rarefied samples. Rarefaction curves, Chao1 and Good’s coverage estimates suggest that the sequencing depth was adequate to capture most of the prokaryotic diversity in each sample (Supplementary Figure 3). Of the total number of OTUs, 464 (representing 0.052% of the total number of sequences) were unique to Butha-Buthe, 363 (0.05%) were unique to Thaba-Tseka, 440 (0.07%) were unique to Mafeteng, 373 (0.06%) were unique to Mokhotlong, 319 (0.10%) were unique to Maseru, and 439 (84.43%) were shared between all the locations (Supplementary Figure 4).

**FIGURE 2:**
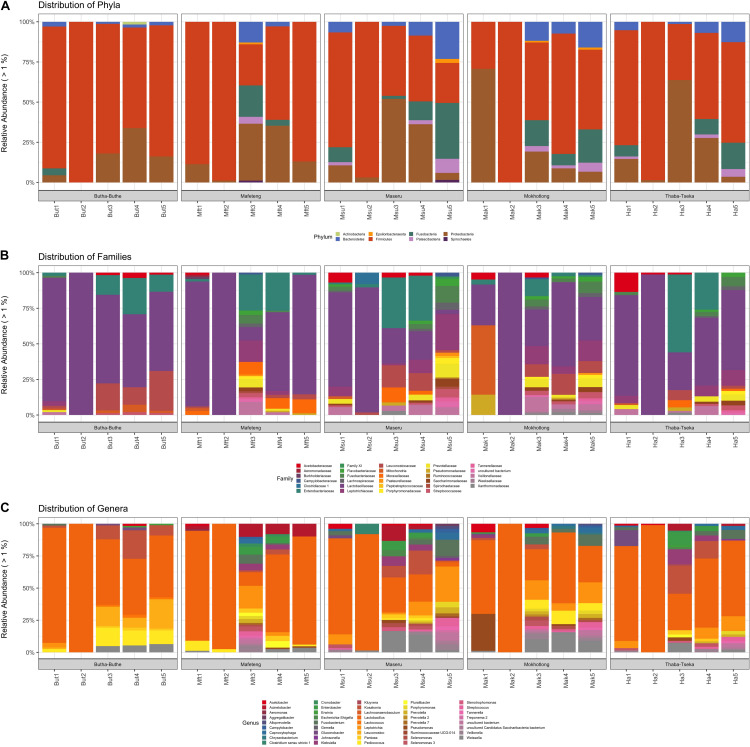
Distribution of bacterial taxa in *Sesotho*. **(A)** Phylum, **(B)** Family, and **(C)** Genus level.

Proteobacteria and Firmicutes were the dominating phyla in all samples and locations (Figure 2A). Overall an increase of Proteobacteria is observed at stage 3 of all brewing processes, most likely due to the addition of extra grains, more started and the interaction of the brewer with the process at this point. However, a decrease in Proteobacteria and an increase in Firmicutes is observed from brew steps 3 to 5 as fermentation progressed. Proteobacteria is the major phylum of Gram-negative bacteria and they include a wide variety of human and soil-borne microbiota, as well as pathogenic and spoilage microorganisms. On the other hand, Firmicutes are the major group of Gram-positive bacteria and they include a wide variety of LAB. This group of bacteria usually produces fermentation by-products such as bacteriocins, CO_2_ and organic acids that will inhibit the Gram-negative bacteria (Todorov and Dicks, 2005; Mufandaedza et al., 2006; Rhee et al., 2011; Perez et al., 2014). The Proteobacteria present, likely originated from the raw materials used to prepare *Sesotho* as well as the individuals producing the brew. The Firmicutes are also derived from the raw materials as well as from the starter cultures (Hammes et al., 2005). The reduction of Proteobacteria during the fermentation is both expected and desirable, as it improves food safety and protects the beverage from spoilage.

**FIGURE 3:**
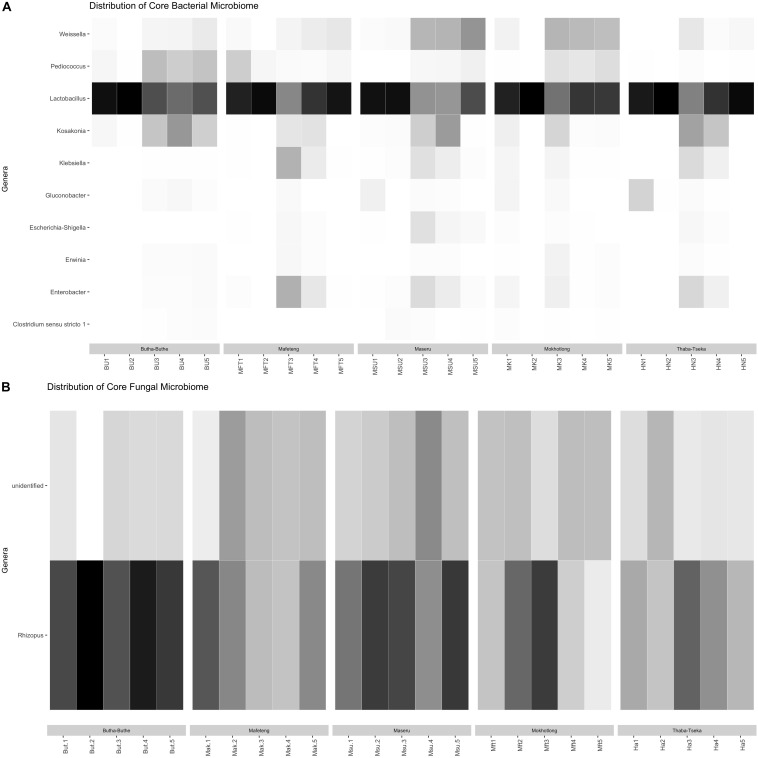
Heatmap of genera within the **(A)** bacterial and **(B)** fungal core microbiomes.

The bacterial families that were the most dominant in all samples are the Lactobacillaceae and Leuconostocaceae of the phylum Firmicutes and the Enterobacteriaceae from the pylum Proteobacteria (Figure 2B). All stages of the brewing process were dominated by the LAB (e.g., genera *Lactobacillus*, *Lactococcus*, *Leuconostoc*, *Pediococcus*, *Streptococcus*, and *Weisella*; Figure 2C), but especially the 2nd stage of fermentation. The core bacterial microbiome, identified across all locations and brew steps was also dominated by LAB (Figure 3), with *Lactobacillus* being the dominant member. These core members highlight the importance of the LAB in the production of *Sesotho.* The LAB play important roles in fermented food products, such as preservation, flavor as well as nutritional benefits (Chelule et al., 2010a, b; Rathore et al., 2012).

The disappearance of several Gammaproteobacteria during the brewing process as the LAB start to dominate the consortium is of note. A number of important human pathogens such as members of the genera *Salmonella*, *Escherichia coli*, and *Vibrio* (Ben Omar and Ampe, 2000; de Vuyst, 2004; Mufandaedza et al., 2006; Cálix-Lara et al., 2014; Kergourlay et al., 2015) were found in the samples; particularly in the 1st and the 3rd stages of brewing. The introduction of these microorganisms can be attributed to the brewers, as it is at these stages where the brewers add the starter cultures as well as some raw ingredients by hand (i.e., starting raw materials in stage 1 and sorghum malt in stage 3). Some of these are soil-borne bacteria (sourced from the raw ingredients), whereas others probably are harbored by the brewers as part of the normal human microbiota. It is interesting to note that the prevalence of the Gammaproteobacteria was similar in all the analyzed samples from Lesotho, regardless the variation in brewers as well as location.

### Fungal Diversity and Abundance

A total of 46 fungal OTUs (97% similarity cut-off), ranging from 5 to 13 OTUs per sample, were found using identical sequencing depth in all samples. Rarefaction curves, Chao1, and Good’s coverage estimates suggest that this sequencing depth was adequate to capture most of the diversity in each sample (Supplementary Figure 5). Of the total number of OTUs, no OTUs were unique to Butha-Buthe, 5 (representing 0.56% of the total number of sequences) were unique to Thaba-Tseka, 4 OTUs (0.56%) were unique to Mafeteng, 4 (0.56%) were unique to Mokhotlong, 4 (0.56%) were unique to Maseru, and 6 (80.73%) were shared between all the locations (Supplementary Figure 6). The most likely reason for the dramatic loss in reads and OTUs, post rarefying and filtering, is that many fungal OTUs remain unclassified in environmental studies focusing on poorly studied environments (Grossart et al., 2016; Rojas-Jimenez et al., 2017). New species, genera or families often remain unidentified even at kingdom rank if they do not have any reference sequences available in the UNITE database (Heeger et al., 2019). Clearly, fungal diversity can be severely underestimated and underrepresented.

**FIGURE 4:**
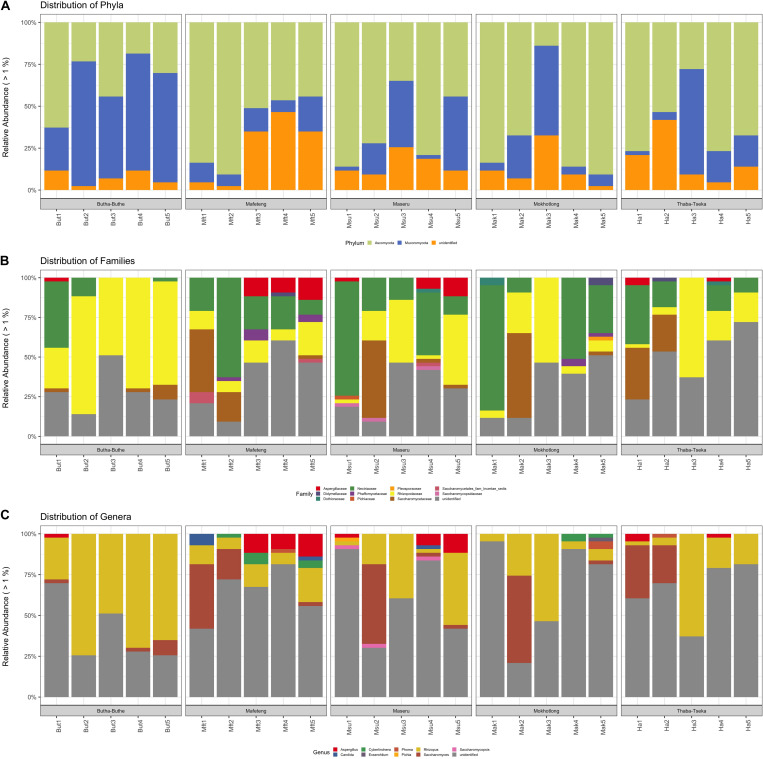
Distribution of fungal taxa in Sesotho. **(A)** Phylum, **(B)** Family, and **(C)** Genus level.

Ascomycota and Mucoromycota were the dominating fungal phyla in all samples and locations (Figure 4A). The phylum Ascomycota play a major role in the production of fermented foods. *Saccharomyces* spp., an example of the Ascomycota, is used in the production of fermented alcoholic beverages such as beer (Walker and Stewart, 2016). The Mucoromycota are a diverse group of molds, most notably the common bread molds, *Rhizopus*, and *Mucor* (Snyder et al., 2018) are common soil fungi (Ziaee et al., 2016). Aidoo and Nout (2010) found that the major role these filamentous molds in traditionally fermented beverages is the production of enzymes and the degradation of anti-nutritive factors. The fungal families that were the most dominant in all samples are the Saccharomycetaceae and Nectriaceae of the phylum Ascomycota and the Rhizopodaceae from the Phylum Mucoromycota (Figure 4B).

Similar to what was found for bacteria, the fungi in the samples are likely to come from both the raw materials used to prepare the product as well as the brewers. *Saccharomyces* spp. appear to be most prevalent in the 1st and 2nd stages of the brewing process, while *Rhizopus* spp. is prevalent throughout (Figure 4C). The core fungal microbiome, identified across all locations and brew steps was also dominated by *Rhizopus* and other unidentified fungi (Figure 3). These unidentified core members highlight how little is known about the fungal microbiome of these traditionally prepared beverages and beckons further study and isolation. As for the known members in the samples, *Saccharomyces* spp., have the ability to ferment the basic sugars obtained from the grains into alcohol and carbon dioxide (Walker and Stewart, 2016) and are also known to produce flavor compounds such as organic acids, aldehydes and esters, which are important in the final characteristics of the product. Therefore, yeasts may contribute to the final taste and flavor of *Sesotho* and not just to alcohol production. In addition, yeasts are known to stimulate lactic acid production by LAB (Kayodé et al., 2011; Oro, 2013). These findings suggest that yeasts in addition to the LAB, play an important role during the production of *Sesotho*.

### Functional Groups

**FIGURE 5:**
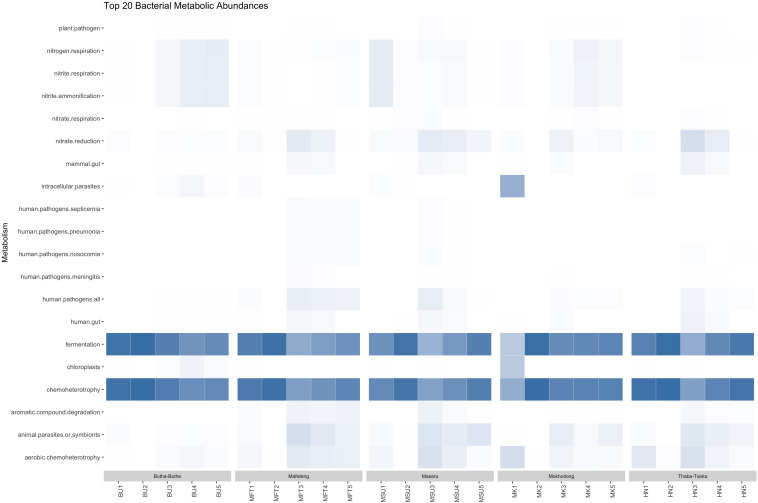
Heatmap of functional diversity of all *Sesotho* bacterial samples during the fermentation process.

Functional annotation, using FAPROTAX, of the 7726 bacterial OTUs revealed a total of 61 metabolic functional groups in the samples (data not shown). Altogether, microbial communities were dominated by chemoheterotrophs and fermentative bacteria (Figure 5). The high level of predicted fermentative metabolism can be attributed to the LAB, such as *Lactobacillus*, *Pediococcus*, and *Weisella*, which were abundant and constituents of the core microbiome. *Lactobacillus* spp. can be involved both in homofermentative metabolism (producing only lactic acid from sugars), or heterofermentative metabolism (producing either alcohol or lactic acid fron sugars; Zaunmüller et al., 2006). Human pathogens, such as *Escherichia* spp., *Shigella* spp., *Klebsiella* spp. and *Stenotrophomonas* spp., appear to be introduced into the samples after the first boiling step, probably due to the second inoculum addition. *Shigella* spp. are among the most important enteric pathogens (causing bacillary dysentery) worldwide (Devanga Ragupathi et al., 2018). *Klebsiella* spp. can cause a wide range of disease states, most notably pneumonia, meningitis, and diarrhea (Podschun and Ullmann, 1998). Some strains of *Stenotrophomonas maltophilia* are known to be pathogenic and has been shown to be a growing source of latent pulmonary infections to individuals who are immunocompromised (McGowan, 2006). Nevertheless, a decrease in these organisms, to less than 1% in all sample but one, is observed as fermentation proceeds. In this regard, some *Pediococcus* spp., which were more abundant at the end of the fermentation process, have shown the ability to inhibit several species of food pathogens (Osmanagaoglu et al., 2001).

### Linking Microbial Community Structure and Functions With the Chemical Profile and Brewery

**FIGURE 6:**
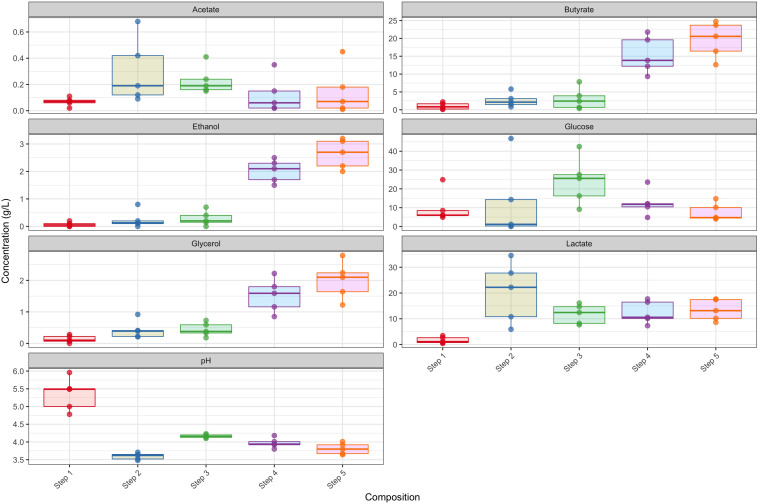
Chemical profiles during the respective steps of the breweries. Each step is representative of the profile of that step in the five different breweries.

The chemical profiles of the respective fermentation stages in the different breweries showed several general trends. Considering steps 1 and 2 as pre-boil and steps 3 to 5 as post-boil, after which a second inoculum is added together with new grain, we see an overall increase in ethanol, glycerol and butyrate in the post-biol steps and a substantial decrease in pH in the pre-boil steps (Figure 6). The observed differences in the production of lactate (mean difference of 11.744 *g*.l^–1^, *p* < 0.05) and butyrate (mean difference of 18.61 *g*.l^–1^, *p* < 0.05) were significant. Both lactate and butyrate are produced by LAB, which dominated all breweries during step 2 of the brewing process, thus inferring that the LAB are responsible for the high butyrate and lactate yields (Taiwo, 2009). This coincides with the significant observed decrease in pH at step 2 (mean difference of −1.5, *p* < 0.05), as lactic acid is being formed during the fermentation process. It is this production of lactic acid and the accompanying change in pH that prevents the further growth of unwanted bacterial pathogens, making the product safe to consume and extending its shelf life (Komesu et al., 2017). The drop in pH is similar to what has been observed in the spontaneous fermentation of other grains like maize (Olsen et al., 1995), millet (Lei and Jakobsen, 2004), and sorghum (Kunene et al., 2000; Muyanja et al., 2003).

Glucose, on the other hand, peaked at stage 3, as additional grains were added during the brewing steps, and then decreased as the fermentation approached the final stages. Glucose production comes from the polysaccharide breakdown of raw materials (i.e., flour and malted sorghum) as well as the brewing style or recipe (Hammes et al., 2005; Nout, 2009; Marshall and Mejia-Lorio, 2012; Gänzle, 2014). The decrease in glucose toward the final stages is probably due to yeast propagation as it was utilized toward ethanol production (Kandler, 1983; Khalid, 2011). Heterofermentative LAB will also produce ethanol during maltose fermentation, a glucose disaccharide, and a key starch moiety (Lohmeier-Vogel et al., 1986). An increase in ethanol is observed during the post-boil steps and the final product appears to be a beer moderately low in ethanol (∼3%; Figure 6). Local consumers measure the “strength” of the beer in bitterness and sourness. A more bitter and sour beer is believed to be more potent (information obtained from conversations with customers). The microbial processes observed in the production of *Sesotho* are very similar to those of the sorghum beers produced by Bantu tribes of South Africa (Taylor, 2003). These *Bantu* beers usually consist of malted sorghum fermented in two stages: a lactic fermentation followed by alcoholic fermentation with *Lb. fermentum* as the dominating LAB species (Sawadogo-Lingani et al., 2007).

**FIGURE 7:**
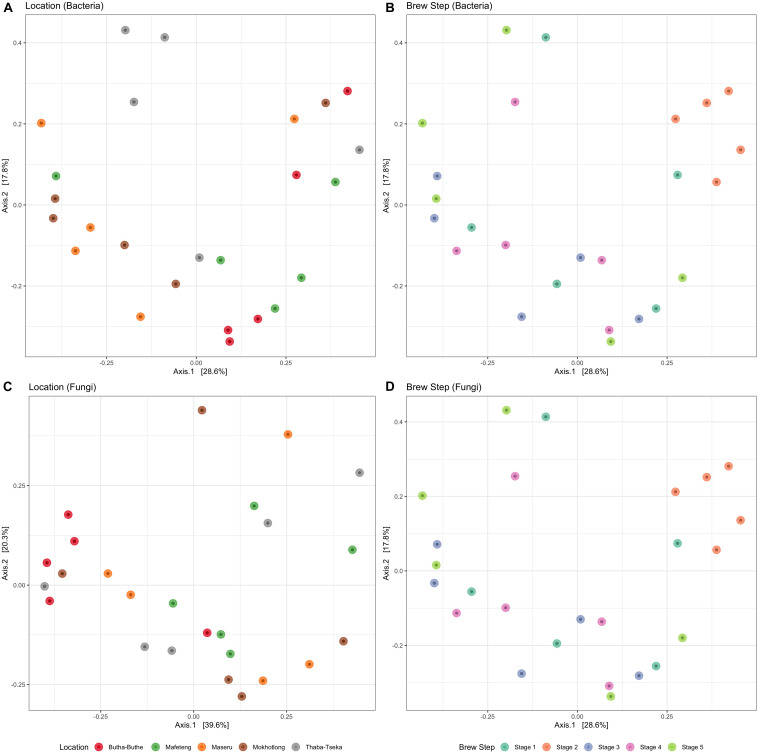
PCoA plots for *Sesotho* bacterial **(A,B)** and fungal **(C,D)** communities, grouping by location **(A,C)**, and brew step **(B,D)**.

Overall, fermentation step was more important than location in explaining structural differences among both bacterial (Figures 7A,B) and fungal communities (Figures 7C,D). Distinct bacterial communities (OTU level) were detected between the five fermentation steps (PERMANOVA *F*_4,2__4_ = 2.51, *R*^2^ = 33%, and *P* = 0.001) using normalized unweighted UniFrac dissimilarities. For example, the clustering of the bacterial communities in the 2nd stage reveals that the structure of those communities was quite similar despite the difference in geographical origin of the brews. This fermentation stage was dominated by the LAB (mostly *Lactobacillus*) and this is also the stage that yielded more lactate production as well as the initial decrease in pH (Figure 6). Neither fermentation step nor location significantly affected PD or OTU richness (Supplementary Figure 7).

Fungal communities (OTU level) were not significantly different between the five breweries (Figures 7A,B; PERMANOVA *F*_4,24_ = 1.40, *R*^2^ = 22%, and *P* = 0.126). However, significant differences were observed during brew steps (PERMANOVA *F*_4,24_ = 2.33, *R*^2^ = 9%, and *P* < 0.05). Although no clustering is evident, in contrast to the bacterial communities, it is clear that dominances in fungal communities shifts during the brewing process in all breweries from *Saccharomyces* spp., to *Rhizopus* spp. and unidentified fungi. The dominance of the mold communities coincides with the increased production of glucose in step 3, as they are responsible for the enzymatic breakdown and hydrolysis of starch to glucose through the production of α-amylases and amyloglucosidases (Nout and Aidoo, 2002).

## Conclusion

The consumption and production of traditionally fermented foods and beverages is in decline due to changes in lifestyle (from traditional to commercial food consumption) due to the effect of globalization. There is thus a scientific and cultural obligation to conserve the habits, recipes, and microbial diversity associated with traditional fermented foods and beverages. These traditional fermentations represent a cultural heritage that can be increasingly valuable in an age where we are moving away from chemical enhancers and preservatives. In addition, fermented products can contribute to the livelihoods of rural communities, through enhanced food security, and income generation via small-scale enterprises. In this work, we reported for the first time the bacterial and fungal diversity through the fermentation process of *Sesotho* and how the final product is shaped by shifts in microbial communities during the brewing process. *Sesotho* showed itself as a hub of unidentified fungi that require further isolation and identification to elucidate their value and potential in food microbiology. In general, the process seems to involved alcoholic, lactic and acetic acid fermentation in the presence of filamentous molds, alcohol-producing yeasts and LAB. During the acidification phase, LAB together with *Saccharomyces*, introduced by traditional brewing methods, appear to play a significant role in the sensorial properties of Sesotho. The dominance of LAB also results in the reduction of harmful pathogens as well as food spoilage organisms. Overall this results in a beer with a unique flavor profile.

## Data Availability Statement

The datasets generated for this study can be found in the NCBI SRA, Project IDs PRJNA605088 and PRJNA605302.

## Author Contributions

EC contributed to experimental design, data analysis, sampling, manuscript writing, and editing and funding. BM was involved in experimental work, data analysis, sampling, and manuscript writing. J-GV helped with the experimental design, sampling, manuscript writing, and editing. MT contributed to experimental design, manuscript writing, and editing. GA was responsible for the experimental design, manuscript writing, and editing. OS contributed to experimental design, sampling, manuscript writing, and editing and funding. MV did the experimental work, manuscript writing, and editing. LS did the experimental design. AV worked on the experimental design, manuscript writing, and editing and funding. BV worked on the experimental design, sampling, manuscript writing, and editing and funding. All authors contributed to the article and approved the submitted version.

## Conflict of Interest

The authors declare that the research was conducted in the absence of any commercial or financial relationships that could be construed as a potential conflict of interest.

## Footnotes

1 https://www.r-project.org/
