# Supplementary figures and images for: Bacterial and Fungal Dynamics During the Fermentation Process of Sesotho, a Traditional Beer of Southern Africa
Source: Front Microbiol. 2020 Jun 30;11:1451. doi: 10.3389/fmicb.2020.01451 (PMC7339052; doi:10.3389/fmicb.2020.01451)

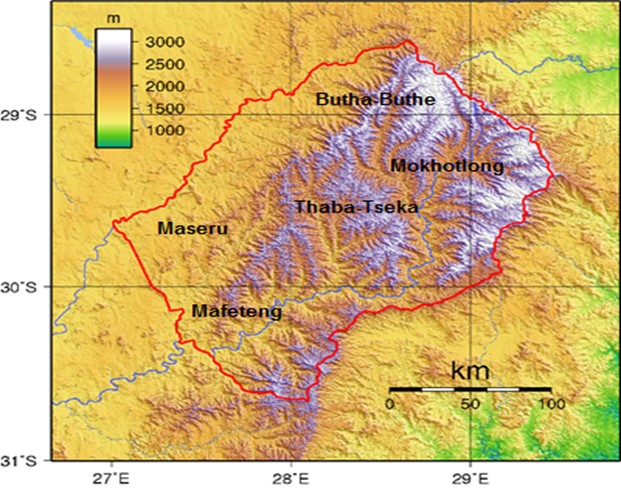

Supplement: Supplementary file 1 [file Image_1.jpg]

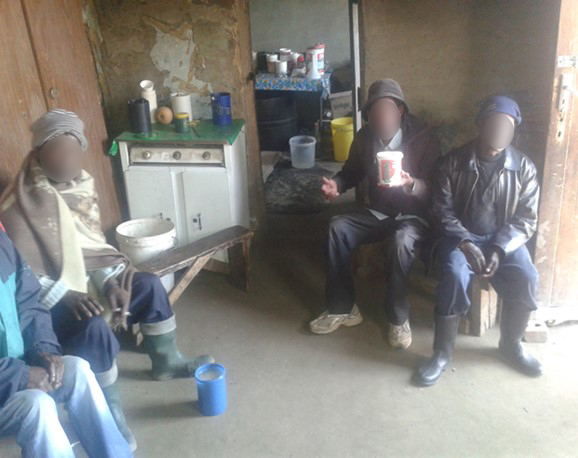

Supplement: Supplementary file 2 [file Image_2.jpg]

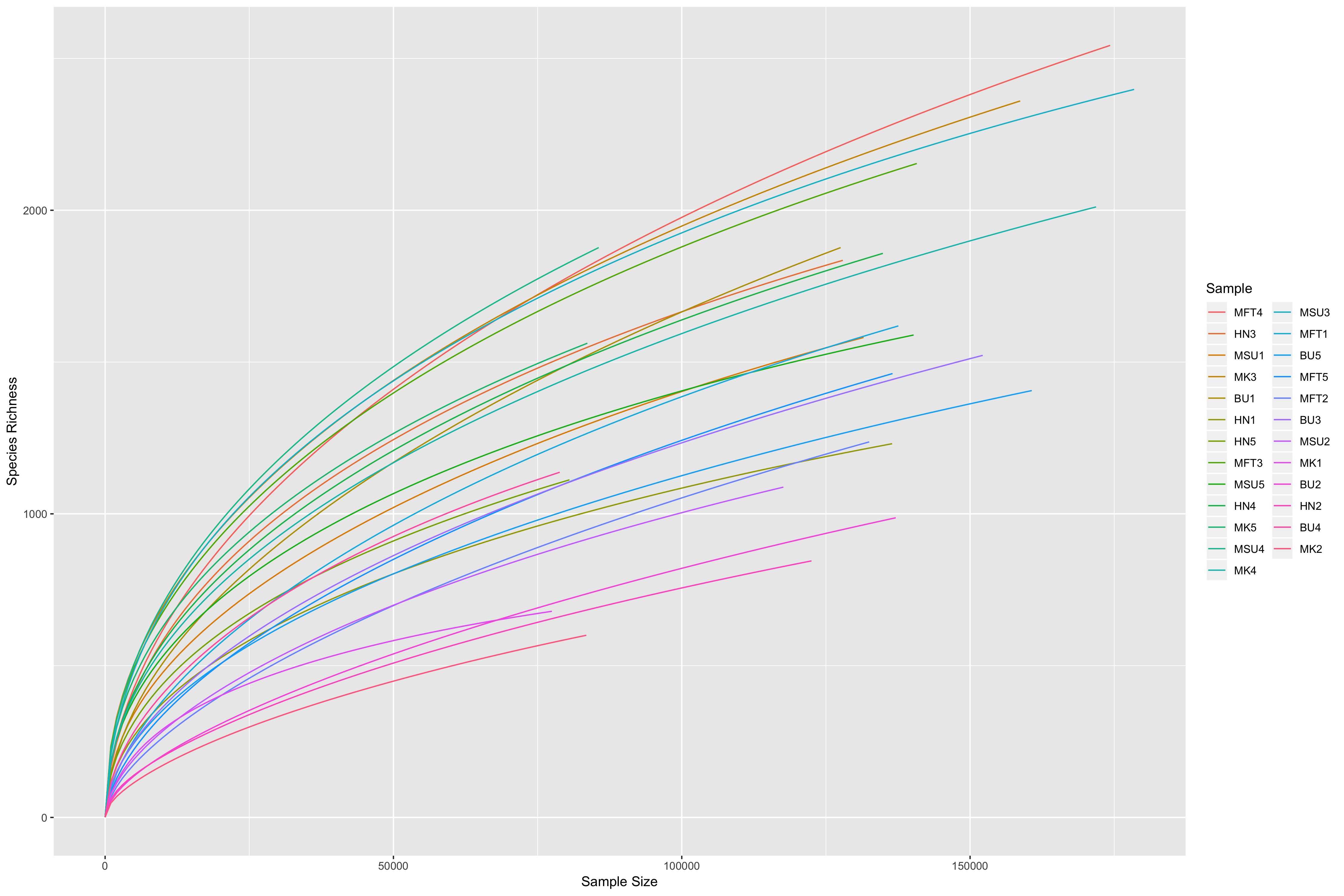

Supplement: Supplementary file 3 [file Image_3.png]

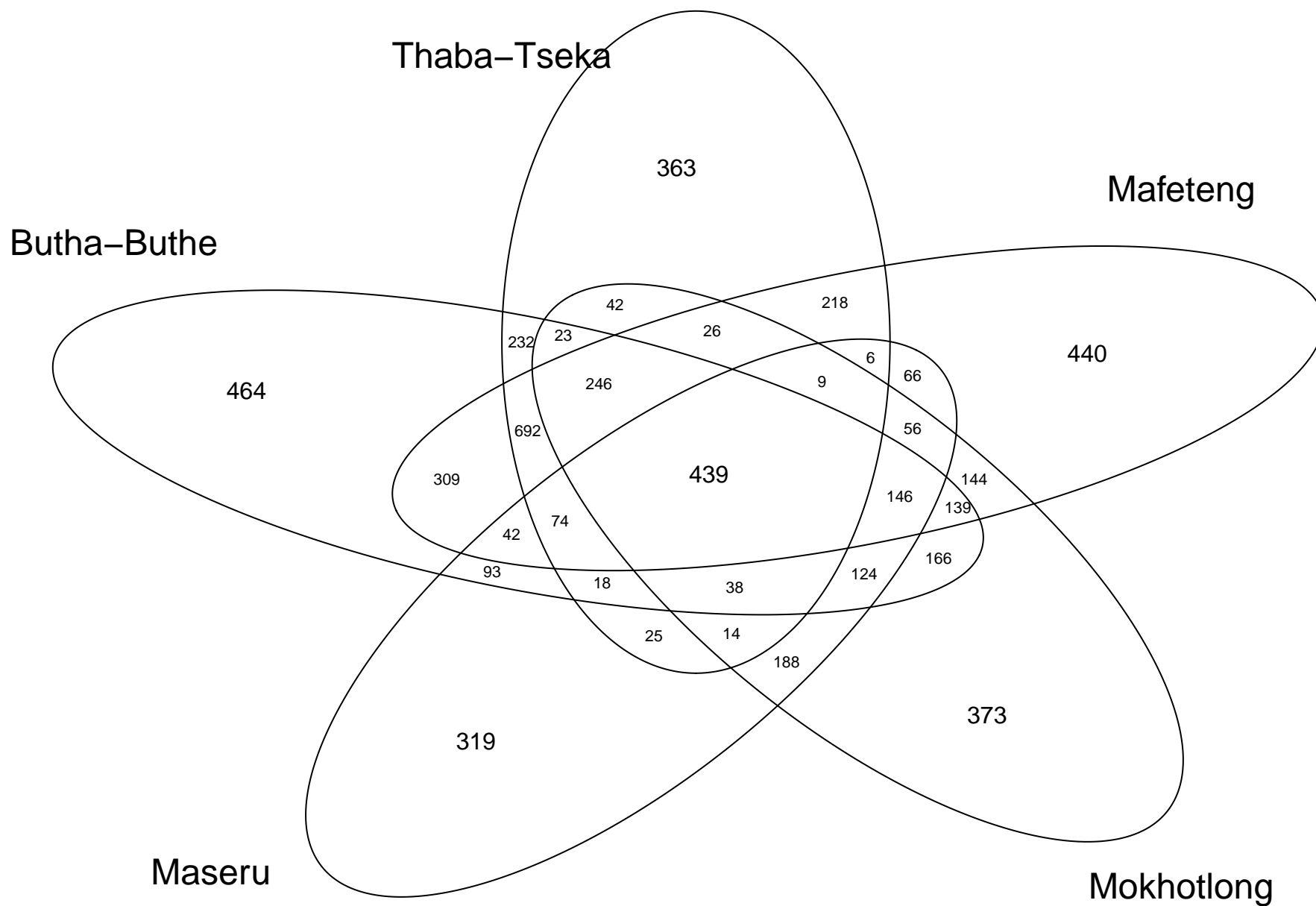

Supplement: Supplementary file 4 [file Image_4.pdf]

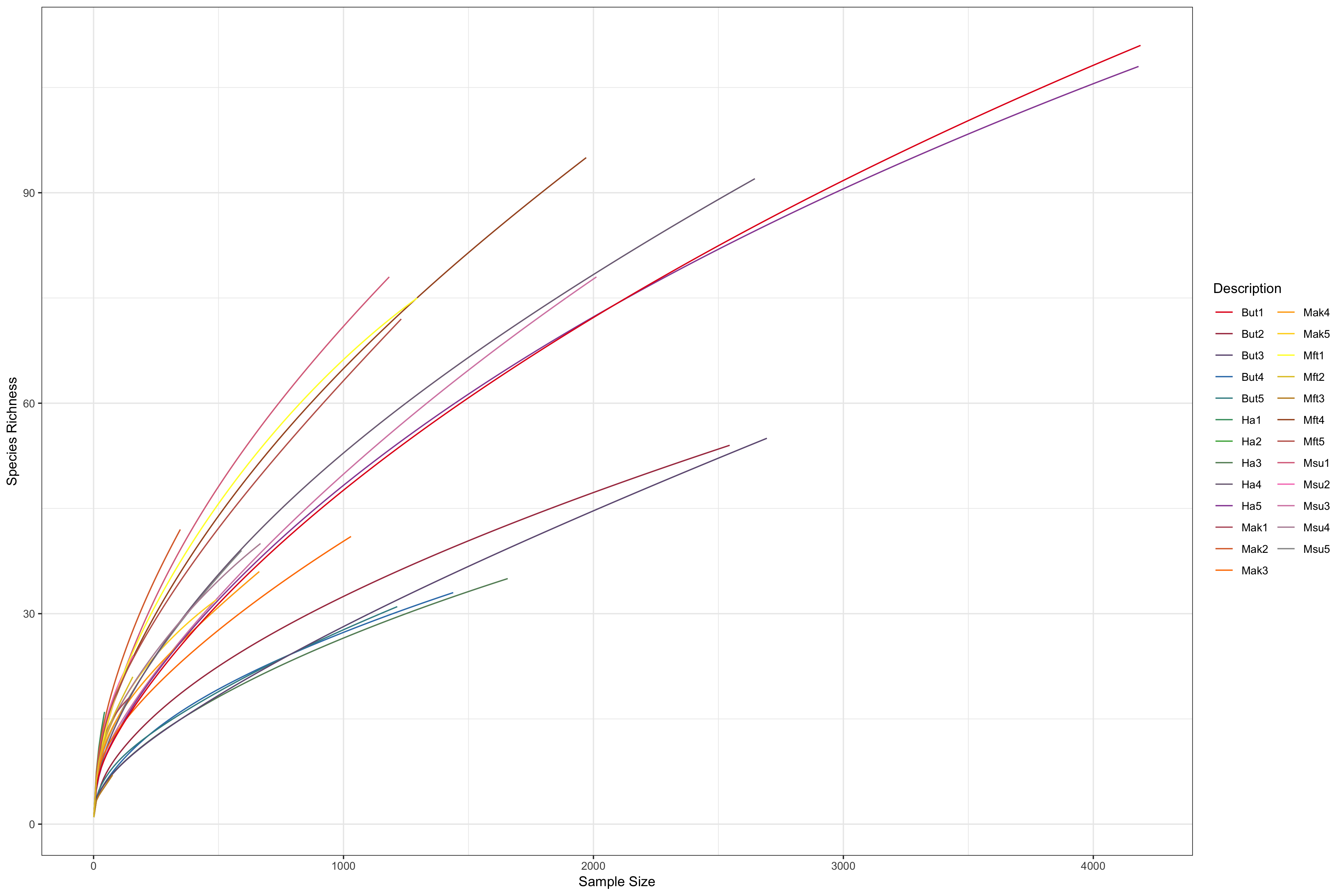

Supplement: Supplementary file 5 [file Image_5.png]

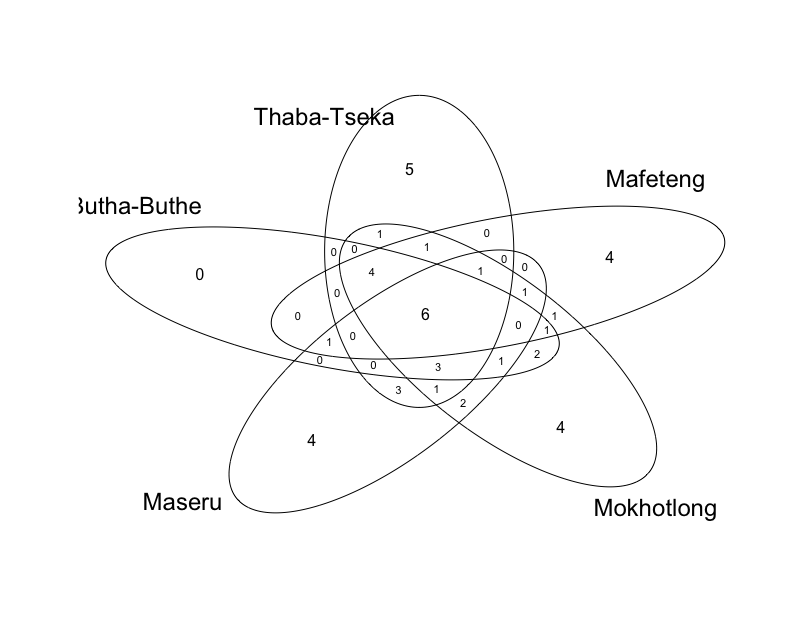

Supplement: Supplementary file 6 [file Image_6.tiff]

Index

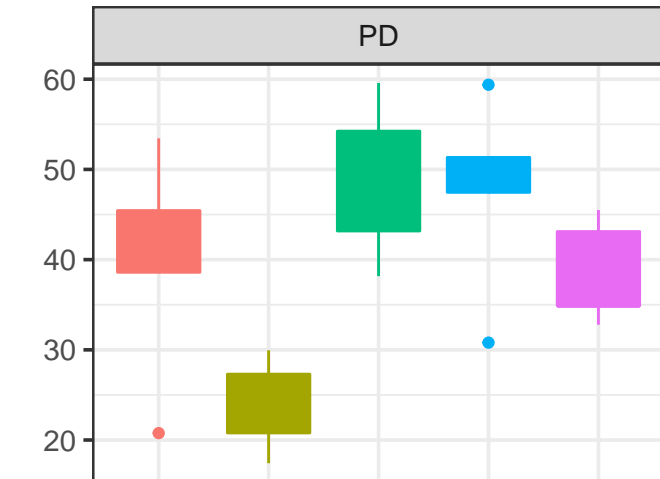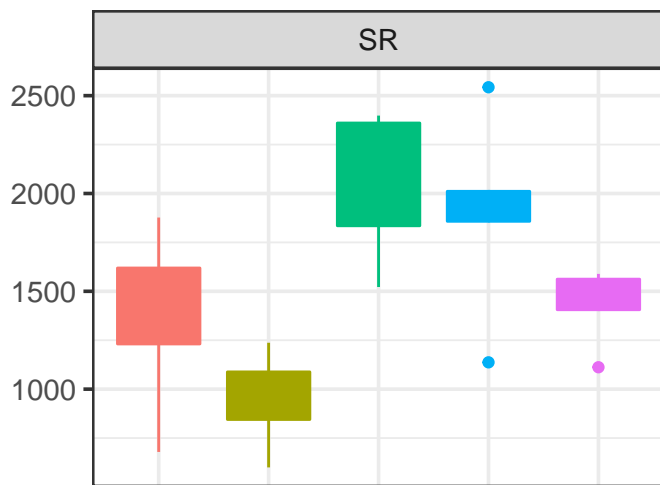

Index

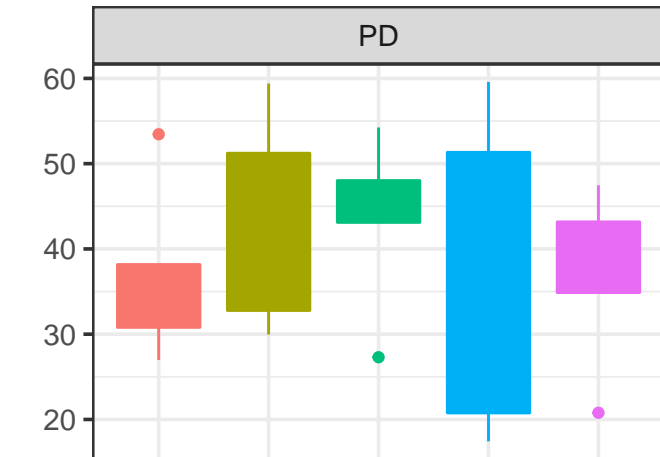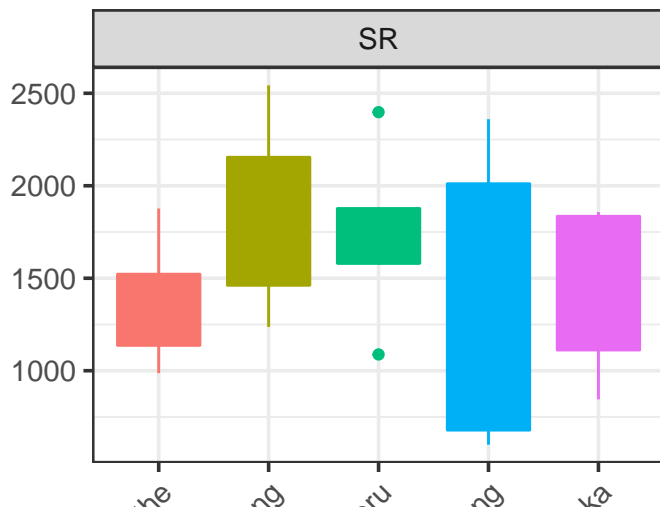

Supplement: Supplementary file 7 [file Image_7.pdf]
