# Supplementary material for: Bacterial and Fungal Dynamics During the Fermentation Process of Sesotho, a Traditional Beer of Southern Africa
Source: Front Microbiol. 2020 Jun 30;11:1451. doi: 10.3389/fmicb.2020.01451 (PMC7339052; doi:10.3389/fmicb.2020.01451)
Supplement: Supplementary file 8 [file Data_Sheet_1.docx]

**Supplementary Figure 1.** A topographical map of Lesotho (Adapted from Mappery).

**Supplementary** **Figure 2.** Local Sesotho brewing households.

**Supplementary Figure 3.** Rarefaction plot indicating the sequence coverage for bacterial sequences.

**Supplementary Figure 4.** Venn diagram of bacterial OTUs shared between different locations.

**Supplementary Figure 5.** Rarefaction plot indicating the sequence coverage for fungal sequences.

**Supplementary Figure 6.** Venn diagram of fungal OTUs shared between different locations.

**Supplementary Figure 7.** Phylogenetic diversities and richness between breweries and brew steps.
