# Supplementary material for: Bacterial and Fungal Dynamics During the Fermentation Process of Sesotho, a Traditional Beer of Southern Africa
Source: Front Microbiol. 2020 Jun 30;11:1451. doi: 10.3389/fmicb.2020.01451 (PMC7339052; doi:10.3389/fmicb.2020.01451)
Supplement: Supplementary file 9 [file Table_1.docx]

**Supplementary Table 1:** Longitude and latitude for sampled sites.

| **Location** | **Latitude** | **Longitude** |
| --- | --- | --- |
| Maseru | 29.29°S | 27.48°E |
| Mafeteng | 29.81°S | 27.53°E |
| Thaba-Tseka | 29.52°S | 28.64°E |
| Butha-Buthe | 28.82°S | 28.51°E |
| Mokhotlong | 29.28°S | 29.13°E |
